# A Unified Pipeline for Simultaneous Brain Tumor Classification and Segmentation Using Fine-Tuned CNN and Residual UNet Architecture

**DOI:** 10.3390/life14091143

**Published:** 2024-09-10

**Authors:** Faisal Alshomrani

**Affiliations:** Department of Diagnostic Radiology Technology, College of Applied Medical Science, Taibah University, Medinah 42353, Saudi Arabia; fshomrani@taibahu.edu.sa

**Keywords:** brain tumor, classification, segmentation, digital healthcare, diagnosis, deep learning

## Abstract

In this paper, I present a comprehensive pipeline integrating a Fine-Tuned Convolutional Neural Network (FT-CNN) and a Residual-UNet (RUNet) architecture for the automated analysis of MRI brain scans. The proposed system addresses the dual challenges of brain tumor classification and segmentation, which are crucial tasks in medical image analysis for precise diagnosis and treatment planning. Initially, the pipeline preprocesses the FigShare brain MRI image dataset, comprising 3064 images, by normalizing and resizing them to achieve uniformity and compatibility with the model. The FT-CNN model then classifies the preprocessed images into distinct tumor types: glioma, meningioma, and pituitary tumor. Following classification, the RUNet model performs pixel-level segmentation to delineate tumor regions within the MRI scans. The FT-CNN leverages the VGG19 architecture, pre-trained on large datasets and fine-tuned for specific tumor classification tasks. Features extracted from MRI images are used to train the FT-CNN, demonstrating robust performance in discriminating between tumor types. Subsequently, the RUNet model, inspired by the U-Net design and enhanced with residual blocks, effectively segments tumors by combining high-resolution spatial information from the encoding path with context-rich features from the bottleneck. My experimental results indicate that the integrated pipeline achieves high accuracy in both classification (96%) and segmentation tasks (98%), showcasing its potential for clinical applications in brain tumor diagnosis. For the classification task, the metrics involved are loss, accuracy, confusion matrix, and classification report, while for the segmentation task, the metrics used are loss, accuracy, Dice coefficient, intersection over union, and Jaccard distance. To further validate the generalizability and robustness of the integrated pipeline, I evaluated the model on two additional datasets. The first dataset consists of 7023 images for classification tasks, expanding to a four-class dataset. The second dataset contains approximately 3929 images for both classification and segmentation tasks, including a binary classification scenario. The model demonstrated robust performance, achieving 95% accuracy on the four-class task and high accuracy (96%) in the binary classification and segmentation tasks, with a Dice coefficient of 95%.

## 1. Introduction

The primary function of the human brain, an essential and intricate organ, is to govern the body. It is in charge of processes like speech, emotional reactions, homeostasis, learning, motor control, cognitive function, consciousness, and sense processing. One of the most frequent cancers brought on by faulty neurons is a brain tumor (BT). This could be either an aggressive malignant carcinogenic tumor or a benign non-carcinogenic tumor that grows slowly [1]. Genetic mutations, immunity disorders, radiation exposure, obesity, and alcohol are a few major causes. According to statistics, the number of people worldwide experiencing brain tumors is rising yearly. This disease has the lowest survival rate; thus, it is crucial to identify, classify, and estimate the duration of the tumor’s existence at initial stages due to its asymmetrical shape, growth rate, texture, and location. As a result, prompt and accurate diagnosis is crucial. Diagnostic procedures such as computed tomography (CT) scan, positron emission tomography (PET), and magnetic resonance imaging (MRI) are used to effectively control factors such as contrast, noise, and boundaries [2]. These imaging techniques aid in the diagnosis of many illnesses. MRI is widely used in the successful diagnosis and treatment of brain cancers because it uses radio waves and safe magnetic fields [3].

Several efficient methodologies and frameworks, as well as segmentation for both qualitative and quantitative analysis, reinforce the significance of the detection of BT [4,5]. Segmentation of medical images is used to perform a basic extract of pertinent information of the area of interest (ROI) [6]. Segmenting brain tumors is essential for analyzing images and scans (such as MRIs, PET, and CTSs) in order to help in diagnosis, treatment planning, and tumor monitoring, for which MRI segmentation is preferred [7,8]. Manual segmentation requires a lot of effort, time, and knowledge. However, as research advances, automated and semi-automatic systems are being created [9,10,11]. Any technique’s adoption in the clinical and pathological domains is contingent only upon the level of user supervision and computation ease. The need for better solutions grows since the manual methods necessitate total user supervision. In practice today and in the future, the semi-automatic or interactive approaches that are already in use will undoubtedly prevail. Radiologists frequently use semi-automated techniques in clinical settings to replace difficult manual processes [12,13].

Emerging machine learning (ML) and soft computing approaches have created a remarkable impact on medical imaging [14,15,16]. PNN (Probabilistic Neural Network), K-NN (K-Nearest Neighbors), ANN (Artificial Neural Network), SVM (Support Vector Machines), and BPNN (Backpropagation Neural Network) are a few BT classification and detection techniques [17,18,19,20,21,22,23,24,25]. These methods work well for classification applications, such as dataset analysis for medical images. Conventional ML systems are linear and work well with smaller datasets, whereas deep learning (DL) approaches, because of their complexity and abstraction, are better at making predictions and drawing conclusions [26,27]. These days, object identification, categorization, and feature extraction are frequently carried out using DL techniques [28]. Recently, many studies have explored DL architecture, its mechanism, performance, and quickness from the perspective of its quantitative utility in MRI-based tumor identification systems as MRI contrast plays a vital role in detection [29]. Datasets usually include images from biopsy, CTS, and MRI.

Convolutional neural network (CNN)-based algorithms have produced dependable results, and are generally regarded as a good technique for semantic picture segmentation [30,31,32]. Despite requiring a sizeable training dataset, CNN-based methods efficiently generate hypotheses and conclusions as they use a self-learning technique [18,33]. In another work, location-specific and channel-agnostic involutional neural networks (InvNets) overcome CNN’s drawbacks like the requirement for intensive parameters (around 4 million), channel specification, and spatial agnostic [34]. The resource-friendly InvNets framework depicted a 92% rate of accuracy efficiently.

Due to its extremely deep hierarchical structure, CNNs, a unique kind of deep learning network, allow for improved latent feature extraction [35]. CNN has proven to be the best at a number of tasks, such as super-resolution, detection, classification, and segmentation [30,36,37,38]. Its segmentation structures vary, especially when it comes to medical images. Among the noteworthy architectures are SENet (Squeeze-And-Excitation Network), ResNeXt, VGGNet (Visual Geometry Group), LeNet-5, U-Net, and DenseNet [35]. The particular objective, dataset, and computing limitations are generally taken into consideration while selecting an architecture. Researchers are always looking for ways to make CNNs more effective, accurate, and interpretable for a wider range of uses. By identifying latent correlations between undersampled and fully sampled k-space data for MRI reconstruction, Wang et al. became the first to utilize CNNs [39].

By reusing the alternate direction method of multipliers (ADMM), which was first applied for CS-MRI reconstruction techniques, Yang et al. enhanced the network architectures even further [40]. Schlemper et al. created a cascaded framework for the more focused reconstruction of dynamic sequences in cardiac magnetic resonance imaging [41]. Zhu et al. created a unique framework to give more dense mapping over domains via its suggested automated transform by manifold approximation, allowing for additional latent mapping in the reconstruction model [42]. Guinea et al. proposed an optimized denoised bias correction model that effectively segments low-contrast, intensity-inhomogeneous images by incorporating denoising and bias correction into the active contour model, outperforming traditional methods and deep learning approaches in terms of accuracy and speed [43].

While numerous efficient methods exist for detecting and outlining BT, manual segmentation remains a time-consuming and expertise-intensive task. Automated and semi-automated systems have emerged to address these challenges, but their clinical and pathological adoption hinges on user-friendliness and computational efficiency. Current semi-automated tools offer partial relief to radiologists, yet a demand persists for more refined solutions. DL methods excel at extracting insights from vast datasets but require substantial computational resources and large annotated datasets, limiting their widespread application.

This study introduces a novel integrated approach to automate the analysis of MRI brain scans. By combining a Fine-Tuned Convolutional Neural Network (FT-CNN) based on VGG19 for tumor classification (glioma, meningioma, pituitary) and a Residual-UNet (RUNet) for pixel-wise segmentation, I aim to optimize radiologists’ workflows. This pipeline seeks to enhance diagnostic accuracy and efficiency, ultimately improving patient outcomes. The key contributions are as follows:

1. Unified Pipeline Development: Introduce a novel pipeline that seamlessly integrates classification and segmentation tasks for brain tumor analysis, addressing the scarcity of literature concurrently tackling these crucial aspects.
2. Generalized Approach: Demonstrate the versatility of my pipeline by applying it to two distinct brain tumor datasets. This includes a four-class classification dataset and a two-class classification dataset with accompanying segmentation masks, showcasing the adaptability of my approach to various data structures and classification complexities.
3. Comprehensive Performance Evaluation: Through extensive experimentation on multiple datasets, including the Figshare dataset (encompassing meningioma, glioma, and pituitary tumor classes) and the additional two-class dataset, I meticulously evaluate the proposed pipeline’s performance across different classification scenarios and segmentation tasks.
4. Robust Model Assessment: Employ a comprehensive set of evaluation metrics, including precision, recall, F1-score, Dice coefficient, IOU, and Jaccard distance, to provide a thorough assessment of my proposed pipeline’s efficacy in both classification and segmentation tasks. These metrics offer valuable insights into the model’s performance across varying dataset complexities and its ability to accurately delineate tumor regions within medical images.
5. Multi-task Learning Insights: Analyze the performance of my unified approach in handling both classification and segmentation tasks simultaneously, providing insights into the benefits and challenges of multi-task learning in the context of brain tumor analysis.

The remaining paper is organized as follows: Section 2 describes the materials and methods applied for the proposed work. Section 3 explains the results and discussion, and finally, the paper is concluded in the Conclusion section.

## 2. Materials and Methodology

### 2.1. Dataset Description

**Figure 1:**
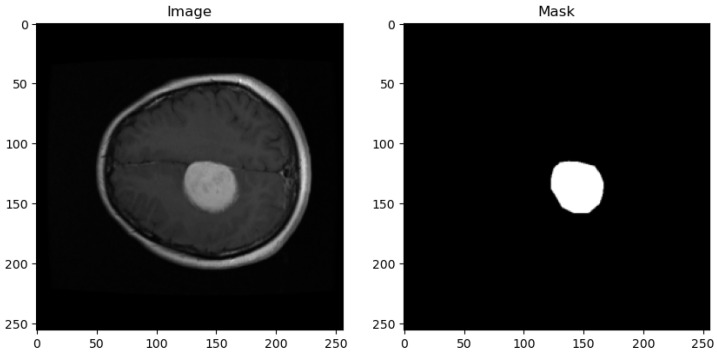
Sample dataset image with corresponding masks.

The dataset utilized in this study is sourced from the publicly available Figshare brain tumor collection. This dataset comprises a total of 3064 T1-weighted Contrast-Enhanced Magnetic Resonance Images (CEMRI) stored in MATLAB (.mat) format. Each image has a resolution of 512 × 512 pixels and represents brain MRI slices obtained from 233 patients. Figure 1 shows the sample image and its corresponding mask from the dataset.

1. Dataset Composition:
  (a) Tumor Types: The Figshare dataset encompasses three distinct categories of brain tumors, namely glioma, meningioma, and pituitary tumor, with varying distributions among the samples.
  (b) Sample Distribution: Among the 3064 MRI images, there are 1426 images corresponding to glioma tumors, 708 images representing meningiomas, and 930 images depicting pituitary tumors, ensuring representation across different tumor types.
  (c) Imaging Modalities: The dataset primarily consists of T1-weighted MRI scans, which are fundamental for visualizing anatomical structures and pathological changes in brain tissue.
2. Dataset Annotation and Structure:
  (a) Annotation Details: Each MRI image entry in the dataset is accompanied by essential information, including the class name (tumor type), patient ID, image data, tumor borders (defined by x and y coordinates outlining various points on the tumor’s boundary), and a binary tumor mask representing the segmented tumor area.
  (b) Data Format: The dataset is stored in MATLAB (.mat) format, facilitating ease of access and compatibility with common programming environments for data analysis and algorithm development.

### 2.2. Dataset Preprocessing

The preprocessing pipeline for the Figshare brain tumor dataset is structured to prepare the data for subsequent analysis and machine learning tasks. Initially, the system imports necessary libraries to facilitate data manipulation and model evaluation. It then defines the directory containing the dataset and specifies the total number of images available, which in this study is 3064. The system initializes an empty list to accumulate information regarding each image, such as its data, label, and associated mask. It subsequently iterates through each image file, stored in a specific format (.mat), extracting the image data and tumor mask using established data access methods. Each image and its mask are resized from their original size of 512 × 512 pixels to a reduced size of 256 × 256 pixels. This resizing step ensures computational efficiency while retaining essential image features for subsequent analysis. Furthermore, the system adjusts the labeling scheme by subtracting 1 from the original labels, aligning them to start from 0. This adjustment conforms to standard indexing conventions commonly used in machine learning tasks. The processed data, including the resized image, resized mask, and adjusted label, are encapsulated within a dictionary for each image and added to the accumulating list.

### 2.3. Feature Extraction

**Figure 2:**
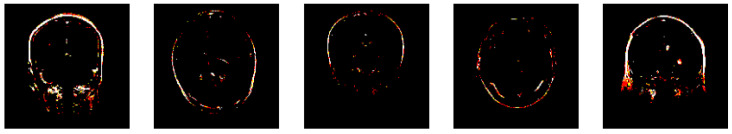
Features extracted by VGG19 layers.

The subsequent code segment serves as a crucial step in the preprocessing pipeline by focusing on feature extraction from the preprocessed brain tumor images. Initially, essential libraries are imported, including train_test_split from sklearn.model_selection for data splitting and components from TensorFlow, such as the VGG19 model and image preprocessing utilities. The images, after undergoing initial preprocessing steps, are resized to match the input size expected by the VGG19 model (256 × 256 pixels) and normalized to pixel values within the range [0, 1] as shown in Figure 2. Concurrently, the corresponding labels for the images are extracted and stored for subsequent analysis.

Following this, the pre-trained VGG19 model is loaded from TensorFlow’s library, with the exclusion of the top classification layer, signifying its utilization for feature extraction rather than classification purposes. Adaptation of the image format is necessary to align with the input shape expected by the VGG19 model, which requires images to be in RGB format. This adaptation is accomplished by duplicating the single grayscale channel to create a 3-channel RGB representation. Subsequently, features are extracted from the preprocessed images using the pre-trained convolutional layers of the VGG19 model.

To facilitate model evaluation, the dataset is then split into training and testing sets using the train_test_split function with an 80:20 ratio from scikit-learn, ensuring that the model’s performance can be assessed on unseen data. The shape of the training labels is (2451), indicating that there are 2451 labels corresponding to the training dataset, aligning with the number of samples. Likewise, the testing labels have a shape of (613), signifying 613 labels corresponding to the testing dataset.

The training features exhibit a shape of (2451, 8, 8, 512), indicating that the training dataset comprises 2451 samples, each represented by an array of shape (8, 8, 512). This shape signifies that each sample has dimensions of 8 × 8 pixels with 512 feature channels extracted by the VGG19 model. Similarly, the testing features have a shape of (613, 8, 8, 512), indicating 613 samples in the testing dataset, with the same pixel dimensions and feature channels as the training set.

### 2.4. Fine-Tuned CNN Model

**Figure 3:**
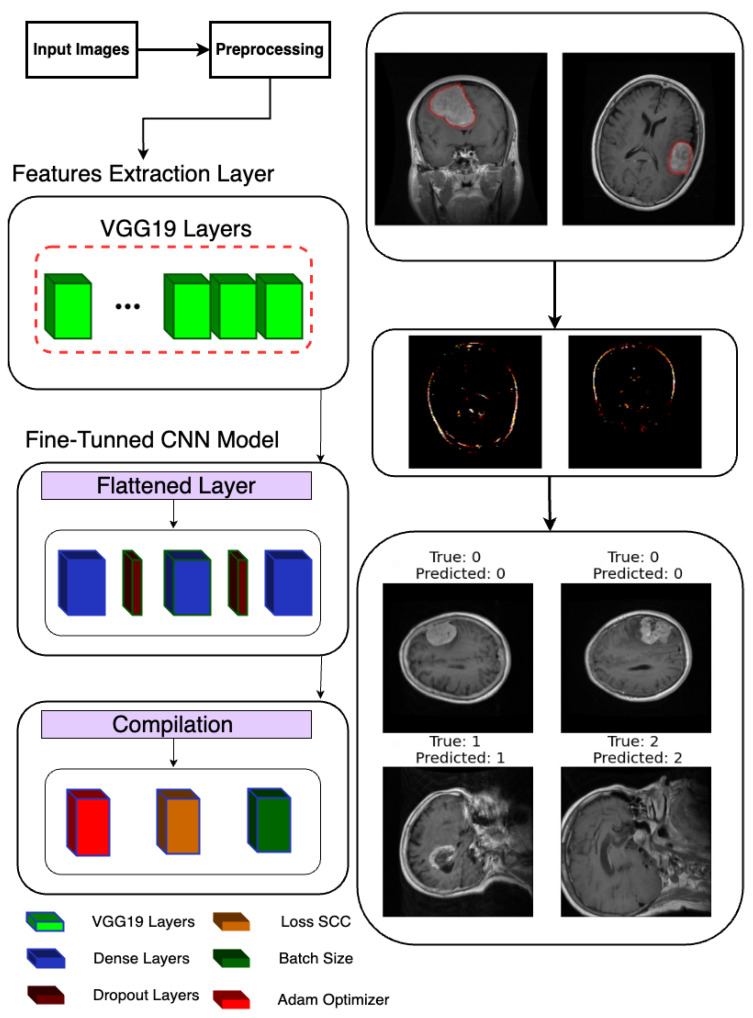
Classification model architecture used in the proposed system.

Following the extraction of features, a deep learning model is constructed and trained to classify brain tumor images as shown in Figure 3. Leveraging TensorFlow’s Keras API, a sequential model is instantiated, denoting a linear stack of layers. The model architecture is defined to process the extracted features, commencing with a Flatten layer designed to flatten the 8 × 8 × 512 feature maps into a one-dimensional array. Subsequently, two densely connected layers with 512 and 1024 units, respectively, are added, each activated by rectified linear unit (ReLU) activation functions to introduce non-linearity. Dropout layers with a dropout rate of 0.2 are interspersed to mitigate overfitting by randomly dropping out 20% of the input units during training.

For the final classification layer, a Dense layer with 3 units and softmax activation is appended to produce probability scores for each of the three tumor classes (glioma, meningioma, and pituitary tumor). This architecture allows the model to predict the likelihood of each class for a given input image. Upon defining the model architecture, it is compiled using the Adam optimizer and sparse categorical cross-entropy loss function, chosen for multi-class classification tasks with integer-encoded labels. Additionally, accuracy is selected as the evaluation metric to monitor model performance during training.

Subsequently, the model is trained using the fit method, where the training data (x_train and y_train) are provided along with the number of training epochs (100), batch size (64), and validation data (x_test and y_test) for evaluating the model’s performance on unseen data. The training process is logged in the history object, allowing for subsequent visualization and analysis of training metrics such as loss and accuracy over epochs. This comprehensive approach to model construction and training ensures robust classification of brain tumor images, with the potential to contribute to accurate diagnosis and treatment planning in clinical settings.

### 2.5. Residual-UNet Architecture

#### 2.5.1. Description of Model Architecture

**Figure 4:**
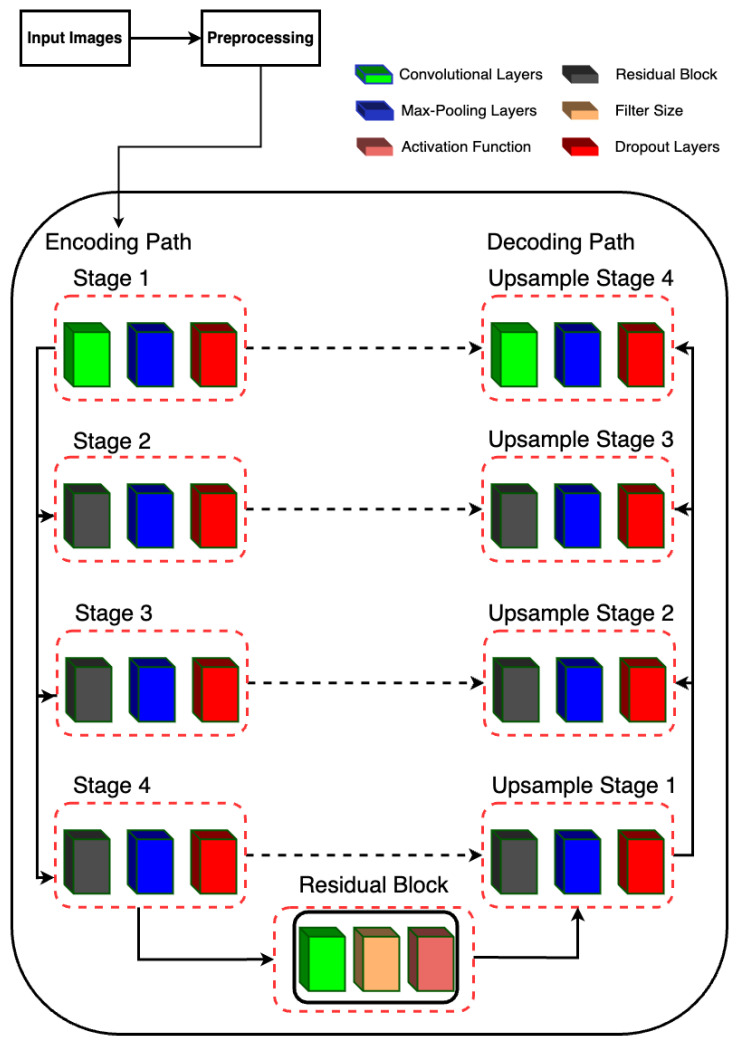
RUNet architecture used in the proposed system.

The proposed model is designed for semantic segmentation tasks, particularly in medical image analysis where precise delineation of anatomical structures is crucial. The architecture follows a U-Net-inspired design, leveraging skip connections to preserve spatial information at different scales. The proposed RUnet architecture is shown in Figure 4.

#### 2.5.2. Encoding Path

The encoding path consists of four stages, each composed of convolutional layers followed by batch normalization, rectified linear unit (ReLU) activation, and max-pooling. Additionally, dropout layers are incorporated after each max-pooling operation to mitigate overfitting.

Stage 1 begins with two consecutive convolutional layers with 16 filters, followed by max-pooling.
Stage 2 utilizes residual blocks with 32 filters, followed by max-pooling.
Stage 3 employs residual blocks with 64 filters, followed by max-pooling.
Stage 4 features residual blocks with 128 filters, followed by max-pooling.

#### 2.5.3. Bottleneck

The bottleneck stage, also known as the bridge, is a critical part of the U-Net architecture. It sits between the encoder and decoder, acting as the deepest layer where the feature maps are the smallest in spatial dimensions but richest in semantic information. In Residual-UNet, the bottleneck stage incorporates a residual block to capture complex features and improve gradient flow during training.

Residual Block (resblock): The residual block applied in the bottleneck stage consists of a series of convolutional layers with residual connections. This design helps in mitigating the vanishing gradient problem, allowing for deeper networks to be trained more effectively. It also facilitates the learning of identity mappings, which are crucial for preserving information across layers. Input and Output: The bottleneck stage takes the output of the fourth pooling layer (pool_4) as its input. By applying the residual block with 256 filters, it produces a feature map that is rich in high-level features. These features are then passed to the decoder for upsampling and reconstruction of the segmentation map.

#### 2.5.4. Decoding Path

The decoding path mirrors the encoding path but involves upsampling operations and concatenation with the corresponding feature maps from the encoding path to recover spatial resolution.

Upsample Stage 1: Upsamples the feature maps from the bottleneck stage and concatenates them with the feature maps from Stage 4. The concatenated feature maps are then processed by a residual block with 128 filters.
Upsample Stage 2: Performs upsampling followed by concatenation with the feature maps from Stage 3. The concatenated feature maps are processed by a residual block with 64 filters.
Upsample Stage 3: Upsamples the feature maps and concatenates with the feature maps from Stage 2, followed by processing through a residual block with 32 filters.
Upsample Stage 4: Finally, upsampling is performed and concatenated with the feature maps from Stage 1, followed by processing through a residual block with 16 filters.

#### 2.5.5. Output Layer

The final output is obtained through a convolutional layer with sigmoid activation, producing a binary segmentation mask.

### 2.6. Model Training

The model is trained using the Adam optimizer with binary cross-entropy loss. Additionally, custom evaluation metrics including accuracy, Dice coefficient, intersection over union, and Jaccard distance are employed to assess segmentation performance. Early stopping is implemented based on validation loss to prevent overfitting during training.

### 2.7. Proposed Pipeline Architecture Flow

**Figure 5:**
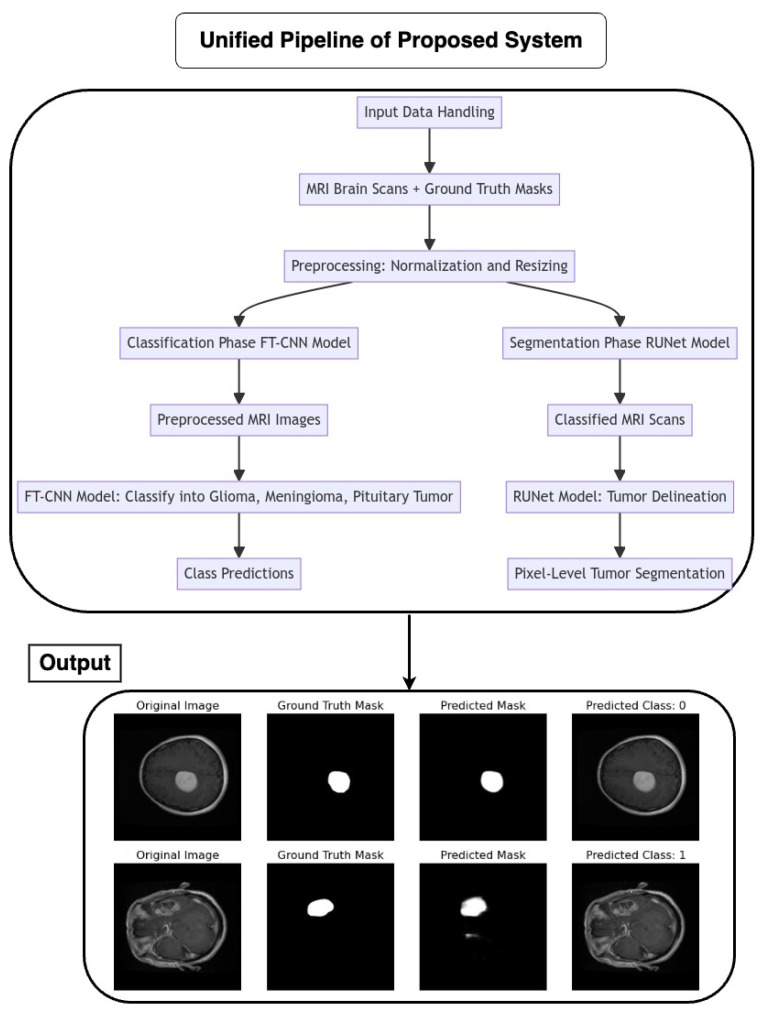
Flow chart of the proposed unified pipeline system.

The pipeline coordinates the seamless integration of an FT-CNN classification model and a RUNet segmentation model for the automated analysis of MRI brain scans as depicted in Figure 5. The flow of the architecture is as follows:

#### 2.7.1. Input Data Handling

MRI brain scans, accompanied by their corresponding ground truth masks, serve as the input data for the pipeline. Preprocessing steps, such as normalization and resizing, ensure the uniformity and compatibility of input images with model requirements.

#### 2.7.2. Classification Phase (FT-CNN Model)

The pipeline initiates with the FT-CNN classification model, which is designed to classify MRI scans into distinct tumor classes: glioma, meningioma, and pituitary tumor. The FT-CNN model processes the preprocessed MRI images and produces class predictions, leveraging learned features to discriminate between different tumor types.

#### 2.7.3. Segmentation Phase (RUNet Model)

Following classification, the pipeline transitions to the RUNet segmentation model for tumor delineation. The RUNet architecture, characterized by a contracting and expanding path, is employed to generate pixel-level predictions, effectively segmenting tumor regions within the MRI scans.

## 3. Results and Discussion

The proposed framework was implemented in Python, utilizing various machine learning and deep learning libraries including TensorFlow, Keras, Pandas, NumPy, and Matplotlib. The framework was evaluated on a system with the following specifications: Nvidia Tesla P100 GPU (1 unit), Quad-core CPU, and 29GB RAM. Due to GPU resource utilization, the computation time per epoch was approximately 95 s for the classification task and around 3 min for the segmentation task. A comprehensive analysis of key performance metrics was conducted to evaluate the efficacy of the proposed model.

For the classification component, the following metrics were assessed: overall accuracy, loss, confusion matrix, classification report, and AUC-ROC curve. These metrics provide insight into the model’s ability to correctly classify the data. For the segmentation component, the following metrics were analyzed: accuracy, loss, Dice coefficient, Jaccard distance, and intersection over union (IoU). These metrics quantify the quality of the segmented output masks generated by the proposed model.

The pipeline of the proposed framework integrates both the classification labels and corrected segmentation masks predicted by the model into a consolidated output frame. This allows for a holistic performance evaluation combining both classification and segmentation results. Overall, the analysis of these key performance metrics on both classification and segmentation tasks provides a quantitative assessment of the strengths and weaknesses of the proposed approach.

Key performance metrics and their corresponding equations are shown in Equations (1)–(9). Accuracy measures the proportion of correct predictions among the total number of predictions made. The loss function measures the error between the actual and predicted values of a model. The classification report provides a summary of evaluation metrics for a classification model, including precision, recall, F1-score, and support for each class. A confusion matrix is a table that summarizes the performance of a classification algorithm. It shows correct and incorrect predictions for each class.

Area Under the Receiver Operating Characteristic Curve (AUC-ROC) measures the performance of a classification model with respect to true positive rate against false positive rate at various threshold settings. The Dice coefficient measures the similarity between two samples. It measures the overlap between predicted and true segmentation masks. Jaccard distance measures dissimilarity between two sets by comparing their intersection and union. It represents a quantitative measure of dissimilarity between segmented masks. IoU measures the overlap between two segmentation masks. It assesses segmentation accuracy by measuring the overlap between predicted and true masks.

(1) $$\mathrm{Accuracy}\;=\;\frac{\mathrm{Number}\;\mathrm{of}\;\mathrm{Correct}\;\mathrm{Predictions}}{\mathrm{Total}\;\mathrm{Number}\;\mathrm{of}\;\mathrm{Predictions}}$$

(2) $$\mathrm{Loss}=\sum_{i=1}^{n}L\left(y_{i},\hat{y_{i}}\right)$$

where $L(y_{i}$, $\hat{y_{i}}$) is the loss function applied to the true label $y_{i}$ and the predicted label $\hat{y_{i}}$.

(3) $$\mathrm{Precision}=\frac{TP}{TP+FP}$$

(4) $$\mathrm{Recall}=\frac{TP}{TP+FN}$$

where TP = true positive, TN = true negative, FN = false negative, and FP = false positive.

(5) $$F1-\mathrm{score}=2\times \frac{\mathrm{Precision}\times \mathrm{Recall}}{\mathrm{Precision}+\mathrm{Recall}}$$

(6) $$Confusion\;Matrix=\left[\begin{array}{cc} TP & FP\\ FN & TN \end{array}\right]$$

(7) $$\mathrm{Dice}\;\mathrm{coefficient}=\frac{2\times \left|A\cap B\right|}{\left|A\right|+\left|B\right|}$$

where *A* and *B* are sets representing the predicted and true segmentation masks.

(8) $$\mathrm{Jaccard}\;\mathrm{distance}=1-\frac{\left|A\cap B\right|}{\left|A\cup B\right|}$$

(9) $$\mathrm{Intersection}\;\mathrm{over}\;\mathrm{Union}=\frac{\left|A\cap B\right|}{\left|A\cup B\right|}$$

**Figure 6:**
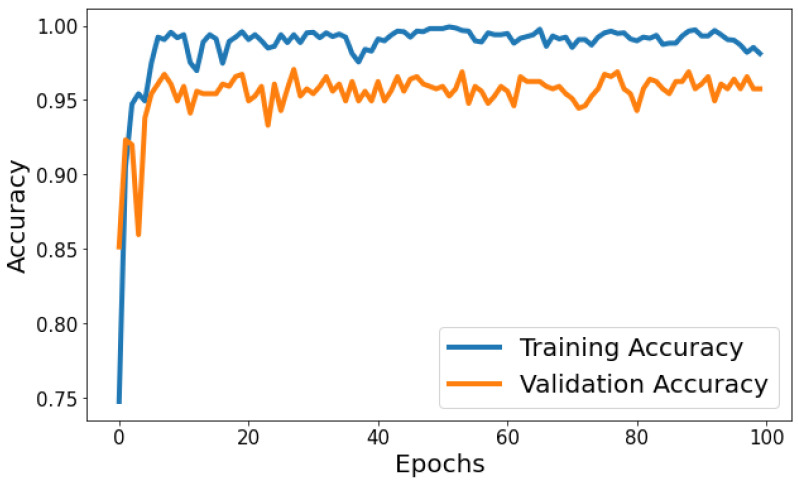
Evolution of training and validation accuracy throughout 100 epochs.

Training Accuracy: Figure 6 shows the behavior of accuracy during the training and validation process. The training accuracy starts at around 74.79% in the first epoch and gradually increases, reaching a peak of approximately 99.80% towards the end of training. Validation Accuracy: The validation accuracy starts at around 85.15% in the first epoch and fluctuates during training. It reaches its highest point of approximately 97.06% around the eighth epoch and generally remains high, although with some fluctuations. Based on Figure 6, the model shows signs of slight overfitting. The consistently higher training accuracy compared to validation accuracy suggests the model is performing better on the data it has seen (training data) than on unseen data (validation set).

**Figure 7:**
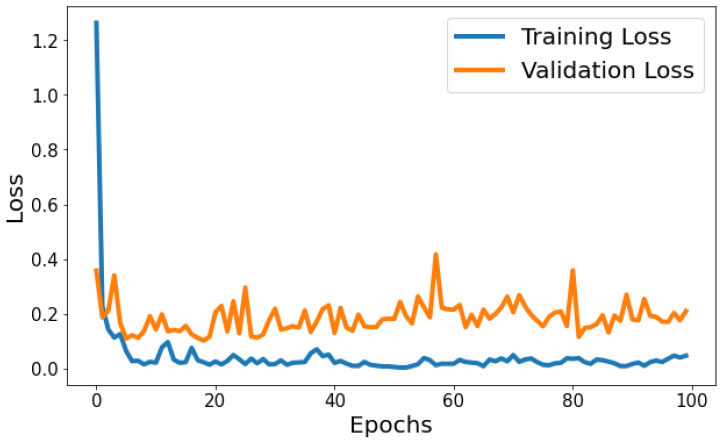
Evolution of training and validation loss throughout 100 epochs.

Training Loss: The training loss starts at 1.2630 and decreases significantly in the initial epochs. It continues to decrease gradually throughout training, indicating that the model is learning and improving its performance. Validation Loss: The validation loss starts at 0.3568 and decreases significantly in the initial epochs, similar to the training loss. It experiences fluctuations throughout training but generally remains relatively low. Figure 7 shows the model loss behavior during the training and validation process. Based on Figure 7, the model shows signs of slight overfitting. The consistently lower training loss compared to validation loss suggests the model is performing better on the data it has seen (training data) than on unseen data (validation set).

Classification Report: In the evaluation of the classification model, several metrics were employed to assess its performance across three distinct classes, denoted as 0, 1, and 2. Precision, recall, and the F1-score were computed for each class, accompanied by a count of actual occurrences known as support. Precision gauges the model’s accuracy in classifying a data point into the correct class, recall measures the model’s capability to identify all relevant instances within a class, and the F1-score provides a harmonic mean of precision and recall, offering a balance between the two in cases of class imbalance.

Table 1 indicates a robust predictive ability across all classes. Class 0 demonstrated a precision of 0.93, a recall of 0.91, and an F1-score of 0.92, with a support of 161 instances. Class 1 exhibited a higher precision of 0.95 and an exceptional recall of 0.98, resulting in an F1-score of 0.97, supported by 269 instances. Class 2 outperformed with a precision of 0.99 and a recall of 0.97, yielding an F1-score of 0.98, with a support of 183 instances.

The overall model accuracy was recorded at 0.96, reflecting the proportion of correct predictions over the total number of cases. Furthermore, the macro average which computes the unweighted mean of each label’s metrics demonstrated high precision, recall, and F1-scores of 0.96, 0.95, and 0.95, respectively. The weighted average, which adjusts the metric based on the true instance count for each label, mirrored the macro average with scores of 0.96 for both precision and recall and an F1-score of 0.96, out of a total of 613 instances. These metrics collectively suggest that the model performs with high accuracy and consistency across all classes.

**Figure 8:**
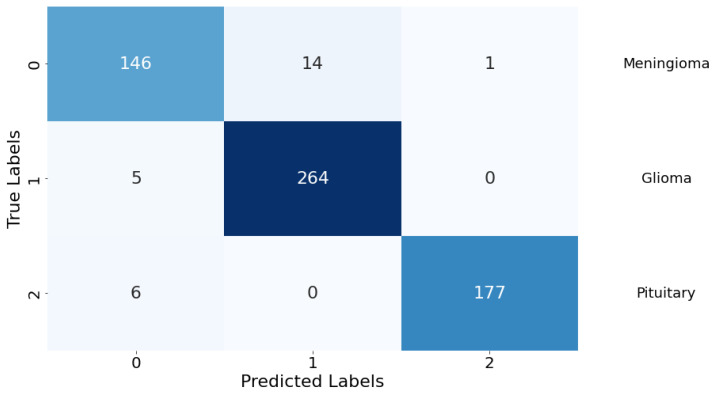
Confusion matrix for the classification model. Rows are true labels, columns are predictions, and diagonal cells show correct classifications.

Confusion Matrix: The classification performance of the model was further elucidated through a confusion matrix, as depicted in Figure 8. The matrix provides a detailed account of the model’s predictive accuracy on a per-class basis. For Class 0 (meningioma), the model correctly predicted 146 instances, while erroneously predicting 14 instances as Class 1 (glioma) and 1 instance as Class 2 (pituitary). Class 1 (glioma) had 264 correct predictions with a minimal misclassification of 5 instances as Class 0 (meningioma). Class 2 (pituitary) demonstrated the highest predictive accuracy with 177 correct predictions, with a marginal misclassification of 6 instances as Class 0 (meningioma). There were no instances of Class 1 (glioma) being misclassified as Class 2 (pituitary), indicating a particularly strong discriminative capability of the model between these two classes.

**Figure 9:**
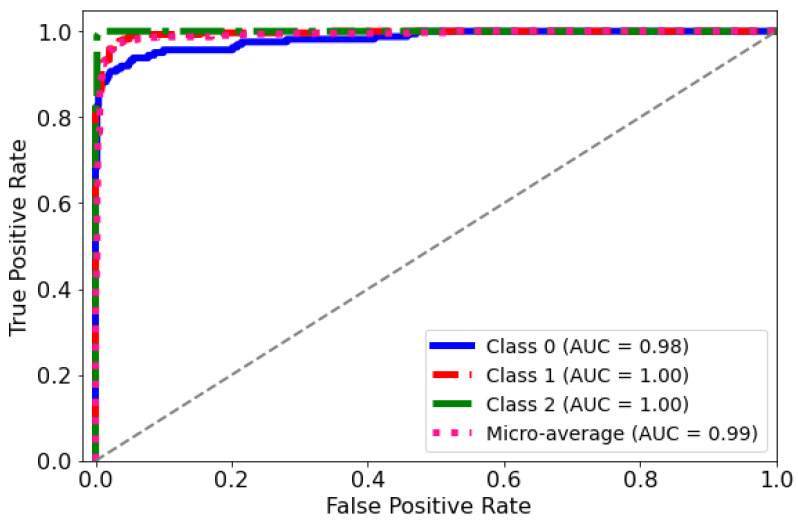
AUC-ROC curve for the classification model. The curve plots the true positive rate against the false positive rate at different classification thresholds.

ROC Curve: The diagnostic abilities of the classification model were further validated through the Receiver Operating Characteristic (ROC) curves for each class, as displayed in Figure 9. The model’s capability to differentiate between the classes was quantified by the Area Under the Curve (AUC) metric for each ROC curve. Class 0 (blue curve) achieved an AUC of 0.98, indicating a near-perfect distinction between true positives and false positives. Classes 1 and 2 (red and green curves, respectively) each achieved an AUC of 1.00, which corresponds to a perfect classification with no misidentified instances. The micro-average ROC curve (dotted line) aggregates the outcomes of all classes and achieves an impressive AUC of 0.99, signifying a superior overall model performance across all thresholds. These ROC curves collectively underscore the high specificity and sensitivity of the predictive model in distinguishing between the different categories of the medical condition under study.

**Figure 10:**
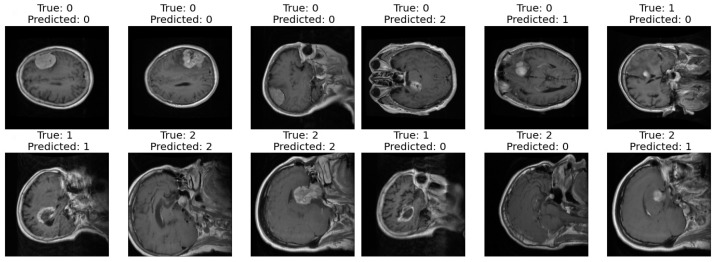
Correct and incorrect sample classification results of the proposed model.

Correct and Incorrect images: In the final step of our analysis, I presented two sets of images to thoroughly evaluate the performance of our model. The first set exclusively showcased images that were correctly classified by our model as shown in Figure 10. Each image was labeled with its true class and the corresponding class predicted by the model. This set provided a clear demonstration of the model’s ability to accurately classify various types of images.

In contrast, the second set exclusively featured images that were incorrectly classified by our model. Similar to the first set, each image in this set was labeled with its true class and the predicted class by the model. This set allowed us to identify specific instances where our model struggled to make accurate classifications, highlighting potential areas for improvement or refinement in future iterations.

Segmentation Results: The presented Table 2 chronicles the progress of a neural network model training across 30 epochs. Each row documents the training and validation metrics attained by the model for the corresponding epoch number. The specific metrics tracked in the analysis include the following: Epoch number (Ep): the training epoch index. Loss: the overall training loss value indicates how well the model fits the training data. Lower values signify better fitting. Accuracy (Acc): the overall accuracy on training data reflects the model’s categorization capability. Higher percentages denote greater predictive accuracy. Dice coefficient (DC): a segmentation evaluation metric applied to training data. Values closer to 1 signify improved segmentation performance. Intersection over union (IOU): another segmentation metric assessing the model’s training segmentation proficiency. Values approaching 1 reflect precise segmentation. Jaccard distance (JD): A further segmentation metric quantifying the disparity between segmented output and ground truth labels on the training data. Values nearer 0 represent accurate segmentation.

The above metrics were also tracked on an unseen validation dataset, prefixed by “V”, at each epoch. Monitoring both training and validation metrics facilitates the assessment of generalization and overfitting tendencies during model optimization.

Examining the table rows reveals the training metrics uniformly improve across successive epochs, loss and Jaccard distance decline while accuracy and other segmentation scores rise. This implies continuous improvements on the trained datasets as optimization progresses. The validation metrics also largely trend better over epochs. However, fluctuations are evident, aligned with the validation data’s unbiased evaluation of generalization capability.

**Figure 11:**
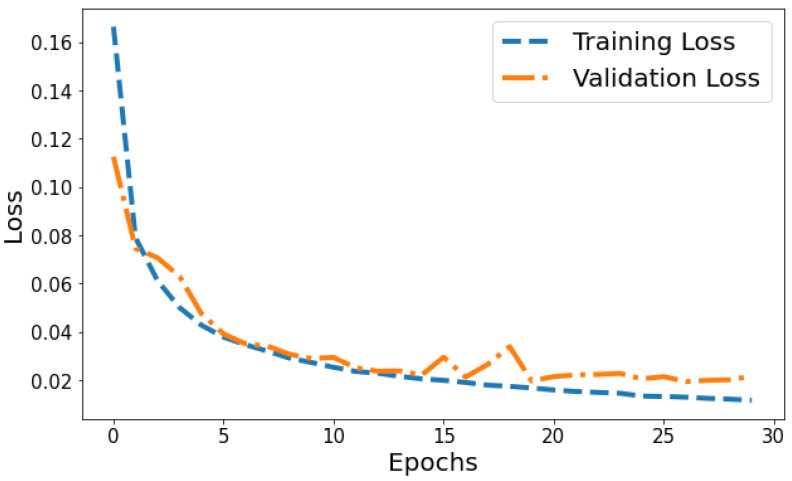
Tracking the training and validation performance of the loss for the proposed model over 30 epochs.

Loss vs Validation loss: A plot of training loss versus validation loss over epochs is shown in Figure 11. The training loss demonstrates a consistent downward trend over epochs, decreasing from 0.1665 to 0.0117 by the 30th epoch. This indicates the model is progressively fitting the patterns in the training data better with each epoch. Meanwhile, the validation loss also generally decreases but shows more fluctuation, ranging between 0.1126 and 0.0339 over training. The overall declining trend suggests that the model’s generalization capability is improving over successive epochs. However, the variability in validation loss compared to the smoothly decreasing training loss plot indicates some slight overfitting. The divergence between training and validation performance is most prominent in later epochs. For example, from epochs 25 to 30, training loss drops from 0.0134 to 0.0117 while validation loss shows a slight increase from 0.0205 to 0.0215. This pronounced gap in late epochs indicates that optimization beyond 25 epochs leads to diminishing generalization performance. Overall, the loss plot suggests good model convergence in early epochs before some overfitting sets in during later epochs.

**Figure 12:**
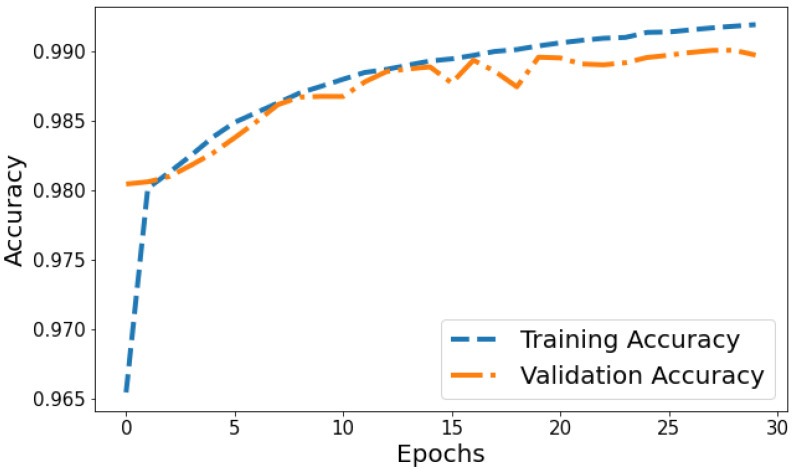
Tracking the training and validation performance of the accuracy for the proposed model over 30 epochs.

Accuracy vs. Validation Accuracy: The accuracy plot in Figure 12 displays the training accuracy steadily increasing from 0.9654 to 0.991 between epochs 1 and 30 as the model fits the training data better. Meanwhile, the validation accuracy fluctuates in the range of 0.9804 to 0.9899, generally trending upwards but at a slower rate than the training accuracy. The gap between training and validation accuracy expands slightly in later epochs; for instance, training accuracy reaches 0.991 in the last epoch compared to a validation accuracy of 0.9897. The overall trends reflect improving model performance on the training data, which translates to moderately better generalization as well. However, validation accuracy plateauing while training accuracy continues increasing indicates potential slight overfitting late in training.

**Figure 13:**
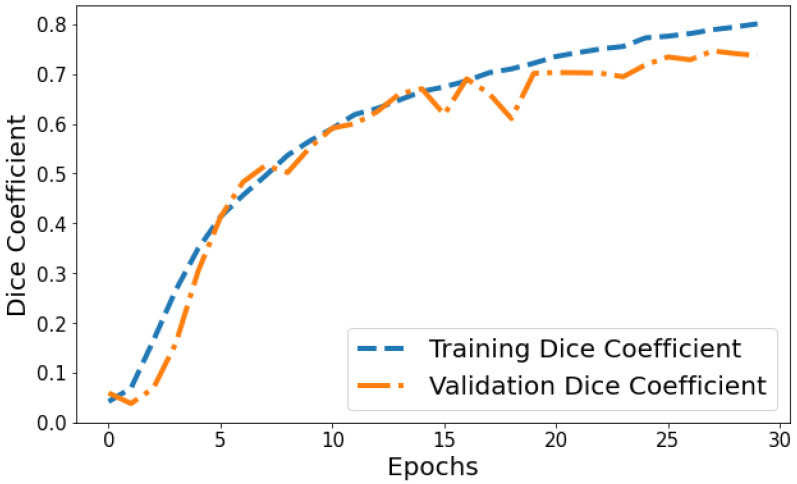
Tracking the training and validation performance of the Dice coefficient for the proposed model over 30 epochs.

Dice Coefficient vs. Validation Dice Coefficient: Figure 13 shows the Dice coefficient plot for both training and validation data over epochs. Training Dice coefficient improves from 0.0419 in epoch 1 to 0.8008 by epoch 30, indicating that the model’s segmentation capability steadily improves on the training data. Validation Dice coefficient also shows an upward trend but is more variable between 0.0591 and 0.7463 over epochs. The gap between training and validation Dice coefficient grows slightly wider in the final 10 epochs, suggesting the model segmentation performance continues enhancing on trained datasets more than general unseen data. But as both training and validation Dice coefficient values are relatively high by epoch 30, it points to good convergence on the segmentation task.

**Figure 14:**
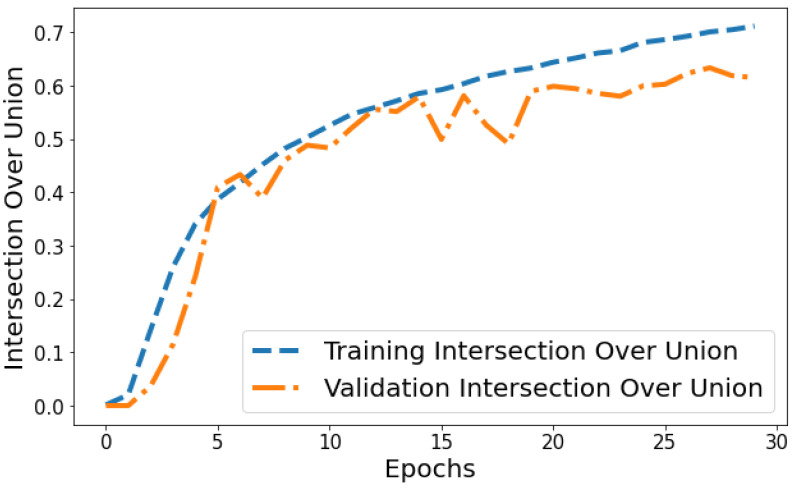
Tracking the training and validation performance of the intersection over union for the proposed model over 30 epochs.

Intersection over Union vs. Validation IOU: The IOU plot in Figure 14 displays the training IOU rising from 0.0014 in epoch 1 to 0.7118 by epoch 30 as the model fits the training patterns better. Validation IOU is more variable, ranging from 0.0000579 to 0.6336 across training. Both training and validation IOU show overall increasing trends, indicating progressively improving segmentation performance on both the trained datasets and unseen data. However, training IOU shows smoother monotonic improvement compared to the more fluctuating validation plot. This suggests some degree of overfitting occurring in later epochs even though generalization capability continues to rise gradually.

**Figure 15:**
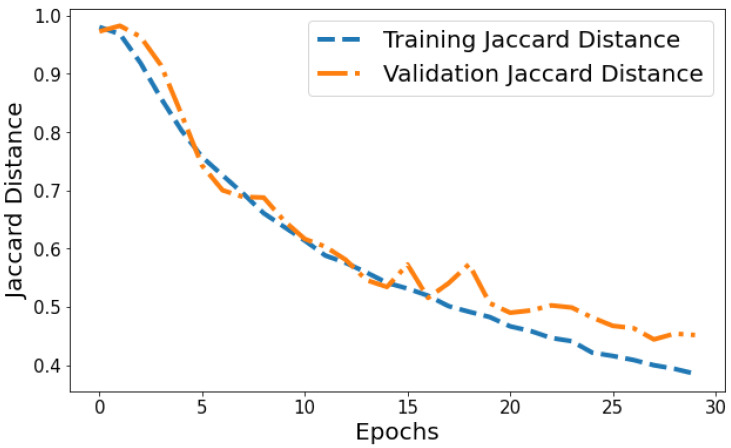
Tracking the training and validation performance of the Jaccard distance for the proposed model over 30 epochs.

Jaccard Distance vs. Validation Jaccard Distance: The Jaccard distance plot shown in Figure 15 displays the model reducing segmentation error on the training and validation data over successive epochs. The training Jaccard distance sees a smooth downward trend from 0.9804 in epoch 1 to 0.385 by the 30th epoch. This indicates the model fits the training patterns progressively better, making fewer segmentation errors. The validation Jaccard distance also shows an overall downward trajectory but is more variable, ranging between 0.9729 and 0.444 across epochs. The gap between training and validation Jaccard distance also expands slightly in later epochs. For instance, epoch 30 training Jaccard distance is 0.385 versus 0.454 validation Jaccard distance. The declining pattern in both plots indicates improving segmentation capability generalized to unseen data as well. However, the validation plot is more volatile with a slight divergence in final epochs. This suggests that continuing training beyond 25 epochs leads to segmentation performance improvements on trained data not fully translating to improved generalization. Nonetheless, the relatively low training and validation Jaccard distance values by epoch 30 reflect suitable convergence of segmentation on both training and test data.

In summary, the training plots display smooth decreasing trends for loss and Jaccard distance, along with increasing accuracy and segmentation metric curves over epochs. This demonstrates progressive model fitting and performance improvements on the trained datasets. The corresponding validation plots also largely show improving patterns, indicating generalization capabilities are also enhancing over training epochs. However, the validation plots are more irregular and volatile compared to the consistent training trends.

**Figure 16:**
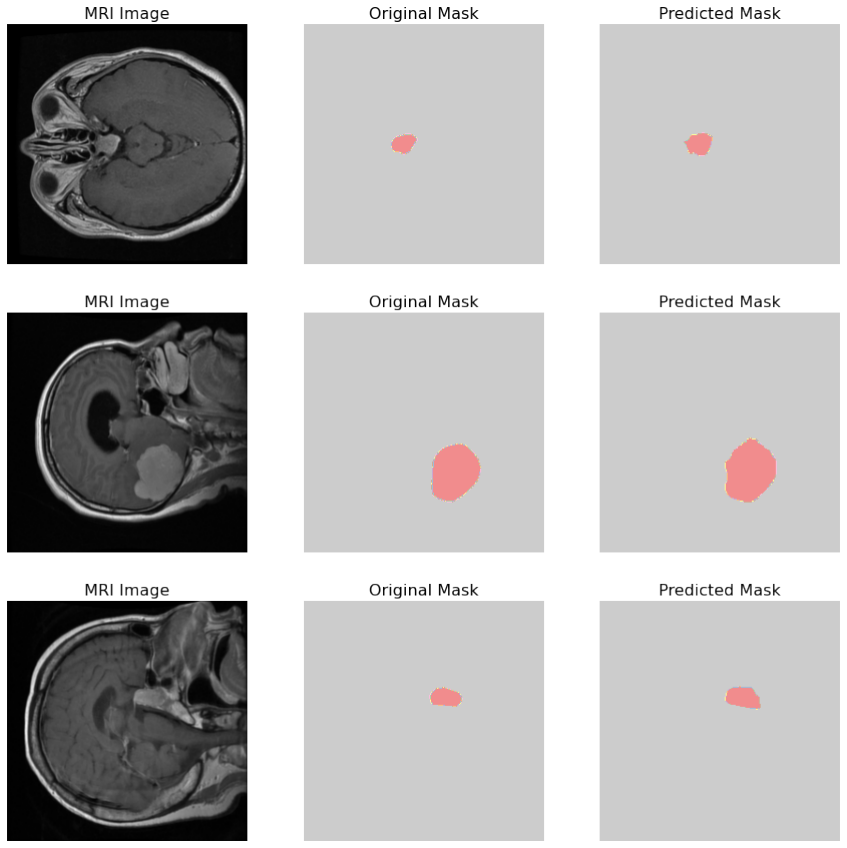
Visual comparison showcasing the original MRI image alongside its corresponding true mask and the predicted mask generated by the proposed model.

The comparative analysis of the original masks and the predicted masks is crucial for validating the machine learning model’s efficacy in medical diagnostics. The objective is for the predicted mask to closely align with the original mask, thereby demonstrating the model’s precision in identifying and localizing pathological features within the MRI scans as shown in Figure 16. 1. MRI Image: Displays the original MRI scan of the patient’s brain, which offers a detailed view of the brain’s anatomy and the potential presence of pathologies. 2. Original Mask: Contains the annotations made by a medical expert. These annotations, highlighted in red, delineate the areas of clinical interest, potentially indicative of abnormalities such as tumors or lesions. 3. Predicted Mask: Showcases the output of a machine learning model trained to identify and demarcate the same regions of interest. These predictions are also highlighted in red and are intended to be compared against the expert’s annotations to evaluate the model’s accuracy.

**Figure 17:**
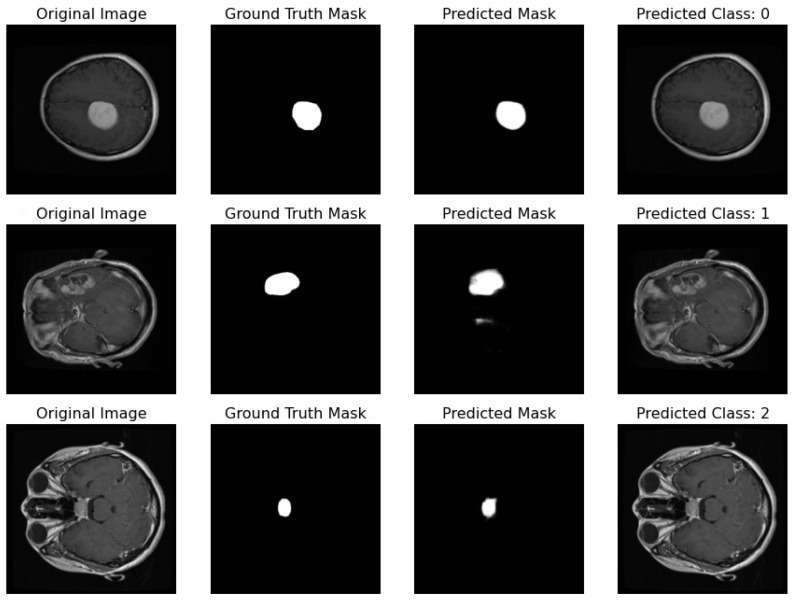
Illustration featuring an MRI image alongside its true mask, predicted mask, and the predicted class label generated by the proposed pipeline.

Proposed Pipeline Performance: I have developed a novel pipeline that can simultaneously perform classification and segmentation tasks on MRI scans. This integrated approach is effectively demonstrated in Figure 17, where each row showcases the dual processing of an individual patient’s MRI scan.

1. Original Image: The pipeline receives the MRI scan as its initial input, showcasing a comprehensive cross-sectional view of the patient’s brain and potential pathological features.
2. Ground Truth Mask: Next to the original image is the ‘Ground Truth Mask’, meticulously annotated by clinical experts to delineate the regions of clinical significance, such as lesions or tumors.
3. Predicted Mask: The segmentation branch of our pipeline then predicts a mask, endeavoring to replicate the expert annotations by encapsulating the region of interest highlighted in the MRI scan.
4. Predicted Class: Simultaneously, the classification branch assigns a ‘Predicted Class’ to the scan. The labels, denoted by integers (0, 1, 2), classify the scan into categories that reflect the model’s interpretation of the underlying pathology.

Our pipeline has successfully integrated the processes of segmentation and classification, enabling concurrent execution with high efficiency. The evaluation of this system is conducted through a comparison between the ‘Predicted Mask’ and ‘Ground Truth Mask’ to assess segmentation fidelity, and the accuracy of the ‘Predicted Class’ against standard diagnostic criteria to validate classification accuracy.

Table 3 presents a comprehensive comparison of several techniques and models utilized for both classification and segmentation tasks, along with their respective datasets and performance metrics. Among the highlighted techniques, the “Proposed model” stands out, employing a combination of Residual-Unet and Fine-Tuned CNN, achieving an impressive classification accuracy of 96% on the Figshare dataset and a segmentation accuracy of 98.8%. Additionally, techniques such as ResNet50-Unet and Fine-Tuned CNN [44], CNN + KNN [45], CNN + SVM [46], and CNNBCN [47] also demonstrate competitive performance, achieving accuracies ranging from 92.6% to 95.82% on various datasets. These datasets include Figshare, TCGA-LGG and TCIA, representing diverse domains such as general image repositories and specific medical imaging challenges. The segmentation results, particularly emphasized by the proposed model, indicate high precision in delineating target objects or regions within images.

### 3.1. Proposed Model Generalization

In this section, I evaluate the generalization capability of our proposed model by testing it on two additional datasets. This evaluation is crucial to demonstrate the robustness and effectiveness of model across diverse data sources.

### 3.2. First Dataset (Classification Task)

Dataset Description: This dataset is a combination of three sources: figshare, SARTAJ dataset, and Br35H. It contains 7023 human brain MRI images classified into four classes: glioma, meningioma, no tumor, and pituitary. The “no tumor” class images were taken from the Br35H dataset. Due to inconsistencies in the glioma class images in the SARTAJ dataset, which affected the results of different models, the data creator replaced these images with those from the figshare site.

**Figure 18:**
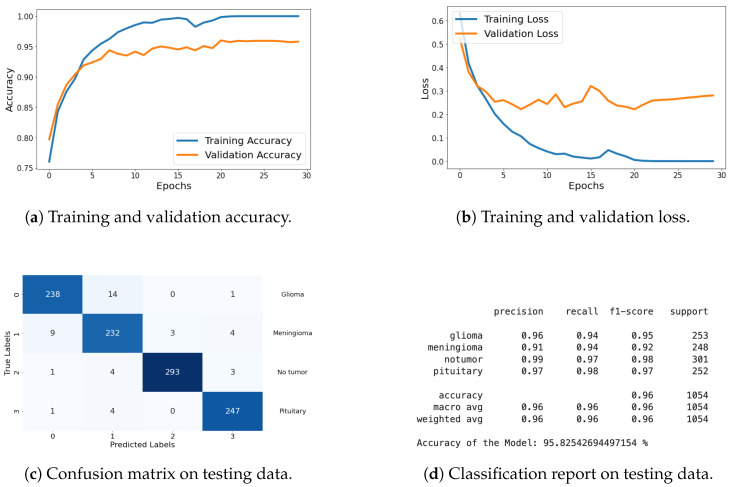
Accuracy, loss, confusion matrix, and classification report of the proposed model in a four-class dataset. The figures represent the training and validation accuracy (**a**), training and validation loss (**b**), confusion matrix (**c**), and classification report (**d**) of the model over 30 epochs.

Classification Metrics: For this dataset, I have included results of loss, accuracy, confusion matrix, and classification report. These metrics demonstrate the model’s effectiveness in accurately classifying the different types of brain tumors. The graphical representation of each metric is shared in Figure 18, and detailed explanations are provided in the previous section.

The results on this dataset demonstrate that proposed model maintains high accuracy and low loss, indicating effective generalization to new data. The confusion matrix and classification report further confirm the model’s ability to accurately classify various categories, with high precision and recall values.

### 3.3. Second Dataset (Classification and Segmentation Task)

The second dataset consists of brain MR images along with manual FLAIR abnormality segmentation masks. These images were sourced from The Cancer Imaging Archive (TCIA) and include data from 110 patients who are part of The Cancer Genome Atlas (TCGA) lower-grade glioma collection, featuring at least fluid-attenuated inversion recovery (FLAIR) sequence and genomic cluster data.

**Figure 19:**
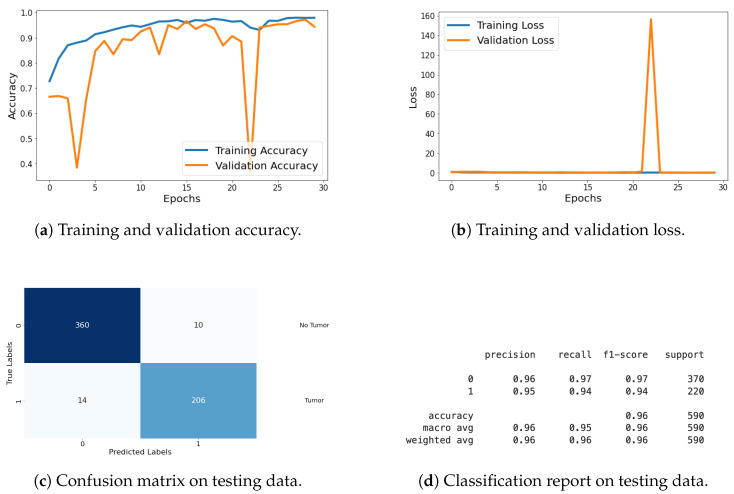
Accuracy, loss, confusion matrix, and classification report of the proposed model in a two-class dataset. The figures represent the training and validation accuracy (**a**), training and validation loss (**b**), confusion matrix (**c**), and classification report (**d**) of the model over 30 epochs.

For the classification task, I evaluated the model using metrics such as loss, accuracy, confusion matrix, and classification report. These metrics provide a thorough assessment of the model’s classification performance, with graphical representations of the metrics being shared in Figure 19. Detailed explanations of these metrics are provided in the previous section.

In the segmentation task, I evaluated the model using various metrics including loss, accuracy (AC), Dice coefficient (DC), intersection over union (IOU), and Jaccard distance (JD) as shown in Table 4. Additionally, I monitored validation metrics such as validation loss (VLoss), validation accuracy (VAcc), validation Dice coefficient (VDC), validation IoU (VIOU), and validation Jaccard distance (VJD) to assess the model’s generalization to the validation dataset. The results of these segmentation metrics are compiled in a table format, showing performance across all 30 epochs. This comprehensive evaluation demonstrates the model’s effectiveness and generalization capabilities in both classification and segmentation tasks.

The results from this dataset illustrate that the model excels in both classification and segmentation tasks. The high accuracy and low loss values in both tasks signify effective learning and generalization. Additionally, the high Dice coefficient, IoU, and low Jaccard distance indicate superior segmentation performance, confirming the model’s capability to generalize well to different types of data.

## 4. Conclusions

In this study, I successfully implemented a novel pipeline for simultaneous brain tumor classification and segmentation tasks, leveraging a Fine-Tuned CNN model and residual UNet architecture. Our proposed pipeline exhibited remarkable performance, with the CNN layers achieving substantial precision, recall, and F1-score values across all tumor classes. Notably, the weighted average accuracy of 96% attests to the effectiveness of our classification approach. Furthermore, for the segmentation task, our model demonstrated significant improvement over epochs, as evidenced by the increasing training Dice coefficient and IOU values, indicating the model’s enhanced segmentation capability and fitting to training patterns. Although validation Dice coefficient and IOU displayed variability, they showcased an overall upward trend, highlighting the robustness of our segmentation model. Moreover, the downward trajectory of Jaccard distance metrics, both for training and validation sets, further corroborates the effectiveness of our segmentation approach. The accuracy plot illustrates the consistent increase in training accuracy, reflecting the model’s improved fitting to the training data, while validation accuracy fluctuates within a narrow range, indicative of the model’s generalization capability. Overall, our study presents a comprehensive framework for concurrent brain tumor classification and segmentation, demonstrating promising results and paving the way for enhanced medical image analysis and diagnosis in clinical settings.

The proposed work has some limitations that should be acknowledged and addressed in future research. First, the current approach utilizes random search for hyperparameter tuning, which, while effective to some extent, may not yield the most optimal parameters for the model. Additionally, the framework is designed to work on a single disease, limiting its applicability to other medical conditions. Another limitation is that the integration of the framework with existing clinical systems has not yet been tested, while the framework is effective in diagnosing the disease, it does not extend to providing treatment or medication recommendations.

## Figures and Tables

**Table 1:** Performance metrics of the proposed classification model.

Class Name	Precision	Recall	F1-Score	Support	Accuracy
0	0.93	0.91	0.92	161	0.96
1	0.95	0.98	0.97	269	
2	0.99	0.97	0.98	183	
Macro avg	0.96	0.95	0.95	613	
Weighted avg	0.96	0.96	0.96	613	

**Table 2:** Summary of performance metrics across epochs for training and validation.

Ep	Loss	Ac	DC	IOU	JD	VLoss	VAcc	VDC	VIOU	VJD
1	0.1665	0.9654	0.0419	0.0014	0.9804	0.1126	0.9804	0.0591	5.79 × 10^−6^	0.9729
2	0.0791	0.9801	0.0688	0.0203	0.9685	0.0746	0.9806	0.0381	3.89 × 10^−5^	0.9826
3	0.0612	0.9813	0.1645	0.1425	0.9189	0.0707	0.981	0.0688	0.0367	0.9638
4	0.0501	0.9825	0.2657	0.2598	0.8581	0.063	0.9818	0.1585	0.1148	0.9147
5	0.0427	0.9838	0.3491	0.3412	0.8028	0.0475	0.9827	0.3037	0.2423	0.8303
6	0.0378	0.9849	0.4129	0.3872	0.7572	0.0391	0.9838	0.4125	0.4096	0.7425
7	0.0347	0.9856	0.4561	0.4183	0.7258	0.035	0.9849	0.4825	0.4335	0.7001
8	0.032	0.9863	0.4952	0.4521	0.6945	0.0341	0.9862	0.5159	0.3894	0.6888
9	0.0291	0.987	0.5367	0.4824	0.6607	0.0307	0.9867	0.502	0.46	0.6876
10	0.0273	0.9875	0.5655	0.5033	0.6371	0.029	0.9867	0.5525	0.4884	0.6476
11	0.0253	0.988	0.5905	0.5254	0.6138	0.0294	0.9867	0.5913	0.4834	0.6165
12	0.0236	0.9885	0.6184	0.5468	0.5881	0.0251	0.9878	0.5999	0.5213	0.6033
13	0.0229	0.9887	0.6307	0.5588	0.5754	0.0237	0.9885	0.6241	0.5559	0.5806
14	0.0215	0.989	0.6477	0.5717	0.5591	0.0238	0.9887	0.66	0.5516	0.546
15	0.0205	0.9893	0.6647	0.5856	0.5412	0.0221	0.9889	0.6702	0.5792	0.5341
16	0.0199	0.9894	0.6739	0.5925	0.5314	0.0295	0.9877	0.6183	0.4993	0.5726
17	0.019	0.9897	0.6861	0.6039	0.5192	0.0212	0.9894	0.6901	0.5814	0.5154
18	0.0179	0.99	0.7024	0.6178	0.5012	0.0265	0.9885	0.6608	0.5263	0.5405
19	0.0174	0.9901	0.7101	0.6266	0.4915	0.0339	0.9874	0.6104	0.491	0.5731
20	0.0167	0.9904	0.7214	0.6331	0.4825	0.0196	0.9896	0.7013	0.5895	0.5063
21	0.0159	0.9906	0.7351	0.644	0.4665	0.0214	0.9895	0.7028	0.5988	0.4898
22	0.0153	0.9908	0.743	0.6519	0.4585	0.0222	0.9891	0.7025	0.5945	0.4938
23	0.0149	0.9909	0.7502	0.6613	0.4465	0.0223	0.989	0.7015	0.5856	0.5025
24	0.0145	0.991	0.7552	0.6658	0.441	0.0228	0.9892	0.6943	0.5803	0.4988
25	0.0134	0.9913	0.7726	0.6812	0.4213	0.0205	0.9895	0.7184	0.5994	0.4816
26	0.0132	0.9914	0.7758	0.6864	0.4156	0.0214	0.9897	0.734	0.6028	0.4673
27	0.0129	0.9915	0.7811	0.6929	0.4087	0.0194	0.9899	0.7284	0.6231	0.4634
28	0.0124	0.9917	0.7889	0.7009	0.3997	0.0199	0.9901	0.7463	0.6336	0.444
29	0.0121	0.9918	0.7942	0.7051	0.3936	0.0201	0.9901	0.7408	0.6188	0.4539
30	0.0117	0.9919	0.8008	0.7118	0.385	0.0215	0.9897	0.7369	0.6151	0.4516

**Table 3:** Comparative analysis of techniques and models for classification and segmentation tasks.

Reference #	Techniques	Dataset	Classification Results	Segmentation Result
Proposed	RUnet and FT CNN	Figshare	96%	98.8%
[44]	Unet and CNN	TCGA-LGG	94%	92%
[45]	CNN + KNN	Figshare	92.6%	-
[46]	CNN + SVM	Figshare	95.82%	-
[47]	CNNBCN	Figshare	95.49%	

**Table 4:** Summary of performance metrics across 30 epochs for training and validation of the proposed model on TCGA dataset.

Ep	Loss	AC	DC	IOU	JD	VLoss	VAcc	VDC	VIOU	VJD
1	0.0788	0.9974	0.9558	0.8802	0.1215	0.1825	0.9934	0.8888	0.7336	0.2674
2	0.0799	0.9974	0.9551	0.8779	0.1237	0.1870	0.9932	0.8874	0.7232	0.2776
3	0.0796	0.9974	0.9552	0.8769	0.1247	0.1843	0.9933	0.8878	0.7152	0.2854
4	0.0785	0.9974	0.9562	0.8803	0.1213	0.1843	0.9931	0.8875	0.7233	0.2775
5	0.0794	0.9974	0.9555	0.8787	0.1229	0.1853	0.9932	0.8872	0.7215	0.2791
6	0.0804	0.9974	0.9548	0.8766	0.1249	0.1877	0.9933	0.8864	0.7373	0.2634
7	0.0793	0.9974	0.9555	0.8787	0.1229	0.1797	0.9931	0.8915	0.7305	0.2704
8	0.0788	0.9974	0.9559	0.8788	0.1231	0.1922	0.9931	0.8827	0.7137	0.2869
9	0.0798	0.9974	0.9552	0.8793	0.1224	0.1834	0.9933	0.8880	0.7215	0.2791
10	0.0797	0.9974	0.9553	0.8786	0.1230	0.1875	0.9932	0.8864	0.7216	0.2792
11	0.0778	0.9974	0.9565	0.8812	0.1207	0.1851	0.9933	0.8867	0.7142	0.2866
12	0.0785	0.9974	0.9562	0.8808	0.1210	0.1845	0.9929	0.8876	0.7140	0.2866
13	0.0784	0.9974	0.9560	0.8790	0.1226	0.1857	0.9932	0.8887	0.7305	0.2701
14	0.0788	0.9974	0.9560	0.8807	0.1211	0.1887	0.9931	0.8849	0.7148	0.2861
15	0.0781	0.9974	0.9562	0.8803	0.1214	0.1908	0.9932	0.8847	0.7278	0.2730
16	0.0791	0.9974	0.9557	0.8800	0.1217	0.1926	0.9931	0.8818	0.7170	0.2838
17	0.0780	0.9975	0.9565	0.8811	0.1206	0.1805	0.9933	0.8920	0.7265	0.2741
18	0.0784	0.9974	0.9563	0.8816	0.1202	0.1840	0.9932	0.8899	0.7314	0.2692
19	0.0775	0.9975	0.9568	0.8816	0.1202	0.1872	0.9931	0.8888	0.7209	0.2796
20	0.0775	0.9975	0.9568	0.8818	0.1200	0.1821	0.9934	0.8919	0.7257	0.2748
21	0.0774	0.9975	0.9570	0.8826	0.1192	0.1960	0.9931	0.8795	0.7272	0.2734
22	0.0795	0.9974	0.9554	0.8799	0.1218	0.1876	0.9931	0.8873	0.7146	0.2857
23	0.0774	0.9975	0.9568	0.8816	0.1202	0.1849	0.9933	0.8895	0.7243	0.2760
24	0.0773	0.9975	0.9568	0.8819	0.1197	0.1778	0.9936	0.8929	0.7219	0.2785
25	0.0773	0.9975	0.9572	0.8810	0.1207	0.1832	0.9934	0.8901	0.7286	0.2719
26	0.0777	0.9975	0.9567	0.8814	0.1203	0.1869	0.9932	0.8892	0.7207	0.2798
27	0.0775	0.9974	0.9566	0.8810	0.1206	0.1946	0.9932	0.8814	0.7127	0.2879
28	0.0775	0.9975	0.9569	0.8826	0.1191	0.1931	0.9933	0.8828	0.7181	0.2822
29	0.0776	0.9975	0.9568	0.8820	0.1197	0.1852	0.9932	0.8884	0.7201	0.2802
30	0.0768	0.9975	0.9574	0.8830	0.1186	0.1812	0.9933	0.8895	0.7268	0.2739

## Data Availability

Data and code can be shared upon request to the author.
